# Pretreatment plasma HGF as potential biomarker for susceptibility to radiation-induced liver dysfunction after radiotherapy

**DOI:** 10.1038/s41698-018-0065-y

**Published:** 2018-10-18

**Authors:** Theodore S. Hong, Clemens Grassberger, Beow Y. Yeap, Wenqing Jiang, Jennifer Y. Wo, Lipika Goyal, Jeffrey W. Clark, Christopher H. Crane, Eugene J. Koay, Simona Dima, Christine E. Eyler, Irinel Popescu, Thomas F. DeLaney, Andrew X. Zhu, Dan G. Duda

**Affiliations:** 1 Department of Radiation Oncology, Massachusetts General Hospital and Harvard Medical School, Boston, MA USA; 2 Department of Medicine, Massachusetts General Hospital and Harvard Medical School, Boston, MA USA; 3 Department of Radiation Oncology, Memorial Sloan Kettering, New York, NY USA; 4 Department of Radiation Oncology, The University of Texas MD Anderson Cancer Center, Houston, TX USA; 5 Center of Digestive Diseases and Liver Transplantation, Fundeni Clinical Institute, Bucharest, Romania

## Abstract

Radiotherapy shows excellent local control in liver cancers but carries the risk of radiation-induced liver dysfunction and liver failure. We conducted a study of plasma hepatocyte growth factor (HGF) in a clinical trial of proton radiotherapy in patients with unresectable liver cancers (NCT00976898), and in an observational study for liver cancer patients undergoing surgical treatments. Liver dysfunction within 3 months after radiotherapy—a Childs−Turcotte−Pugh (CTP) score increase of 1 point or more—occurred in 9/34 (26%) of patients. Patients with no increase in CTP score had lower pretreatment plasma HGF level (*p* = 0.015). Both the increase in CTP score (*p* = 0.034) and the pretreatment plasma HGF (*p* = 0.017) were associated with OS. Plasma HGF was significantly associated with presence of cirrhosis (*p* = 0.0027) and with Model for End-stage Liver Disease (MELD) score (*p* < 0.0001), but not with OS in surgical liver cancer patients. Pretreatment plasma HGF is a candidate biomarker for patient selection for radiotherapy.

## Introduction

Many liver cancers patients are not candidates for curative surgeries because they are not medically fit, have unresectable tumors, or liver transplantation is not feasible. The use of hypofractionated/high-dose radiotherapy has been rapidly increasing for localized disease, because of excellent local control and potential for combination with other therapies.^1–4^ Based on exciting phase II trial results,^3^ we are currently evaluating hypofractionated radiotherapy with protons versus photons in a randomized phase III study (NRG-GI003/NCT03186898). A challenge is that many patients, particularly with hepatocellular carcinoma (HCC), have underlying liver damage and could die due to worsening of hepatic function months post-radiotherapy without disease recurrence. Prior strategies to mitigate hepatic dysfunction have focused on adjusting dosimetric parameters and exclusion of patients with advanced cirrhosis.^2,5^ Other approaches have used imaging biomarkers^6^ and early changes in circulating serum hepatocyte growth factor (HGF)^7^. However, no pretreatment circulating biomarkers are available to predict the susceptibility for worsening of the hepatic function, which would allow selection of cirrhotic liver cancer patients and dose for high-dose radiotherapy.

Blood levels of HGF have been previously associated with increased liver fibrosis and higher Child−Turcotte−Pugh (CTP) score of cirrhosis.^8,9^ HGF is produced primarily by activated stromal cells in the liver and is the ligand to the cell-surface receptor mesenchymal−epithelial transition factor (MET). HGF/Met pathway activation is known to have protective effects on hepatocytes.^10^ However, HGF/MET pathway activation has also been associated with the progression of cancer.^11^ We evaluated the impact of plasma HGF levels on overall survival (OS) and hepatic function after hypofractionated radiotherapy with protons for unresectable liver cancers^3^ and after surgery in resectable liver cancers (see Tables S1-S4).

## Results

**Fig. 1:**
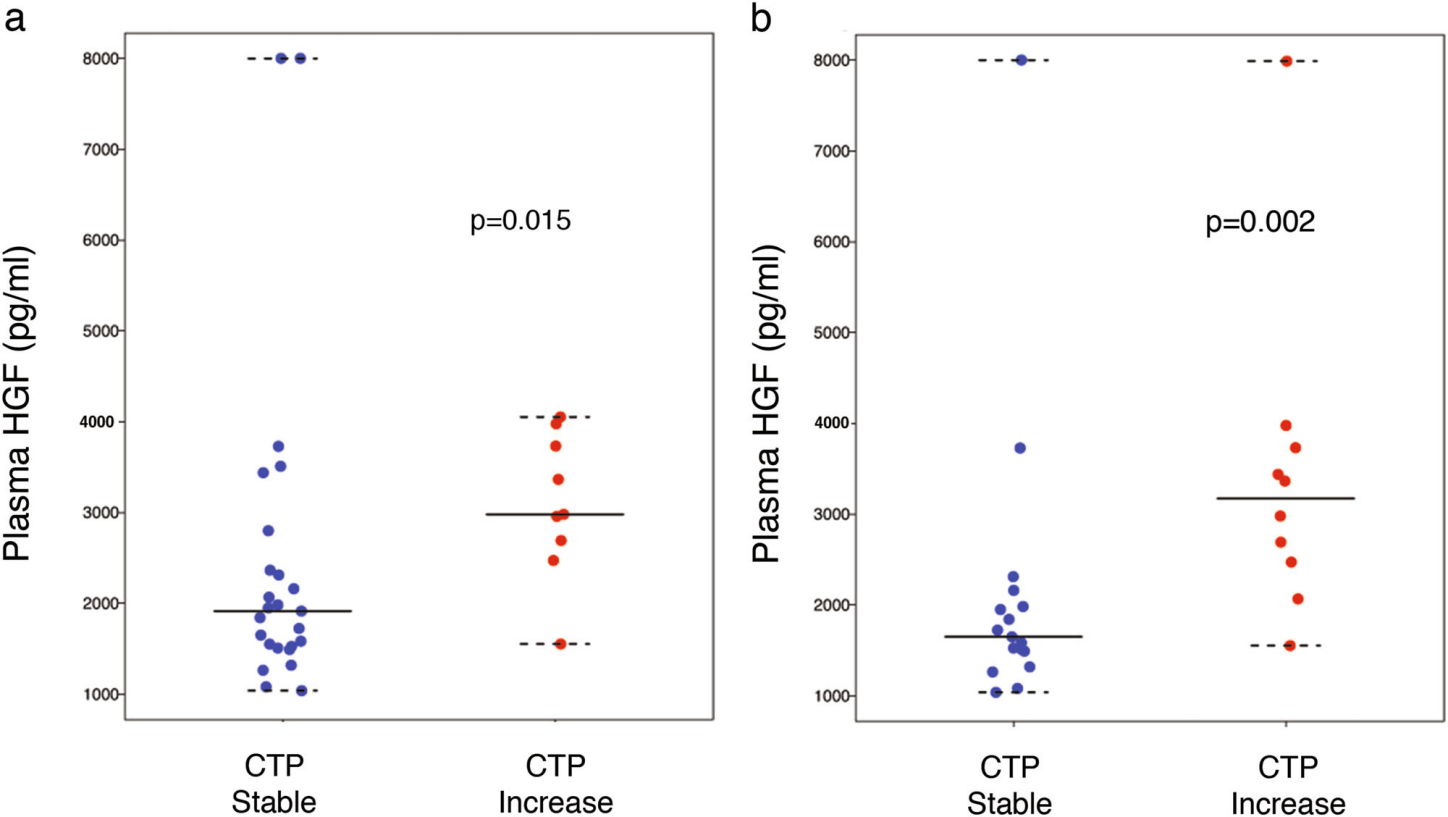
Association between Child−Turcotte−Pugh (CTP) score and pretreatment plasma hepatocyte growth factor (HGF) in liver cancer patients undergoing hypofractionated radiation therapy with protons. **a**, **b** Plasma HGF concentration is significantly higher in patients with worsening in CTP score by at least 1 point after radiation therapy within 3 months **a** and within 6 months **b**. Data shown as median values and ranges

**Fig. 2:**
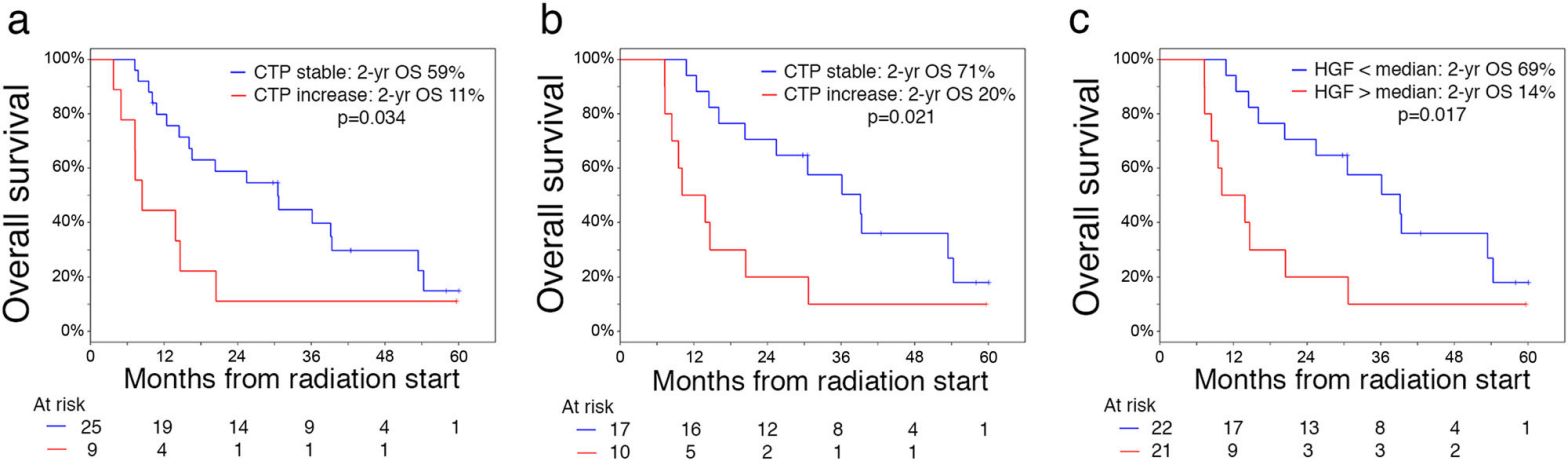
Survival distributions stratified by change in Child−Turcotte−Pugh (CTP) score and plasma hepatocyte growth factor (HGF) in liver cancer patients undergoing hypofractionated radiation therapy with protons. **a**, **b** Overall survival (OS) is significantly and inversely associated with worsening in CTP score by at least 1 point within 3 months **a** and 6 months **b**. **c** OS is significantly and inversely associated with pretreatment plasma HGF level

In the cohort of patients receiving radiotherapy, the median liver tumor size was 5.7 cm and median dose was 58.0 Gy. A CTP increase by ≥1 point occurred in 9/34 (26%) surviving patients at 3 months, including 4/34 (12%) with ≥2-point increase. The median pretreatment plasma HGF concentration was 2311 pg/mL (range 1037–8000) and was comparable between HCC and intrahepatic cholangiocarcinoma (ICC) patients. Patients with no increase in CTP and lower bilirubin had significantly lower pretreatment plasma HGF levels (Wilcoxon rank-sum *p* = 0.01) (Fig. 1a, b and Table S3). While there was no treatment-associated death, patients who had worsening CTP scores had more than twofold the risk of death (log-rank *p* < 0.05) (Fig. 2a, b). Moreover, when stratified at the median value, patients with high plasma HGF had a 2-year OS of 14% compared to 69% in patients with low plasma HGF (log-rank *p* = 0.017) (Fig. 2c), while they showed no difference in progression-free survival (*p* = 0.348). There was no significant change in plasma HGF levels between baseline and day 8 (Wilcoxon signed-rank *p* = 0.545) or 15 (*p* = 0.860) during radiotherapy or between HCC versus ICC patients (Figure S1). Thus, plasma HGF may be a predictive biomarker of susceptibility to radiation-induced liver dysfunction (RILD) and patient survival after radiotherapy.

To delineate the role of HGF in radiation-induced effects versus prognostic value, we next evaluated plasma HGF in surgical HCC patients (*N* = 101). When stratified by the presence of cirrhosis, patients with cirrhosis had significantly higher plasma HGF than those without cirrhosis (*p* = 0.0027) (Table S4). Moreover, plasma HGF strongly associated with Model for End-stage Liver Disease (MELD) score, a validated measure of liver disease severity, in HCC patients undergoing liver transplantation (Spearman rho = 0.54, *p* < 0.0001). However, plasma HGF did not significantly associate with disease-free survival (*p* = 0.22) or OS (*p* = 0.55). These correlations between plasma HGF and cirrhosis and MELD score indicate that pretreatment HGF level can provide additional sensitivity to identify the population vulnerable to hepatic dysfunction with liver failure.

## Discussion

The current individualized radiation treatment for liver cancers is based on mean dose of radiation to the normal liver and is designed to maintain a low risk of classic RILD, a veno-occlusive syndrome. For this reason, classic RILD rates have been low, but nonclassical RILD rates, or worsening hepatic functions, remain high with these same approaches. The current radiation dosing models were not designed to mitigate the risk of nonclassical RILD. In this prospective study, we found a significant association between elevated plasma HGF levels and worsening of CTP score occurring in liver cancer patients treated with high-dose radiotherapy. Importantly, we found that plasma HGF associated not just with the presence of cirrhosis but also with the severity of liver damage in HCC patients. Based on these data, baseline plasma HGF is being currently pursued prospectively as an integral biomarker of liver damage severity and susceptibility to worsening liver function in a phase III trial of radiotherapy for unresectable HCC patients. With successful validation, this study will provide a pretreatment circulating biomarker for patient selection for treatment and radiotherapy personalization.

## Methods

Institutional IRB approval and written informed consent from all patients were obtained before study initiation. Detailed methodology is included as Supplemental Methods.

## Electronic supplementary material

Supplemental Material

## Data Availability

Data are available on request due to privacy restrictions. The data that support the findings of this study are available on request from the corresponding authors T.S.H. and D.G.D. The data are not publicly available due to them containing information that could compromise research participant privacy.

## Electronic supplementary material

**Supplementary information** accompanies the paper on the *npj Precision Oncology* website (10.1038/s41698-018-0065-y).
