# Supplementary material for: Pretreatment plasma HGF as potential biomarker for susceptibility to radiation-induced liver dysfunction after radiotherapy
Source: NPJ Precis Oncol. 2018 Oct 18;2:22. doi: 10.1038/s41698-018-0065-y (PMC6194077; doi:10.1038/s41698-018-0065-y)
Supplement: Supplementary file 1 — Supplemental Material [file 41698_2018_65_MOESM1_ESM.pdf]

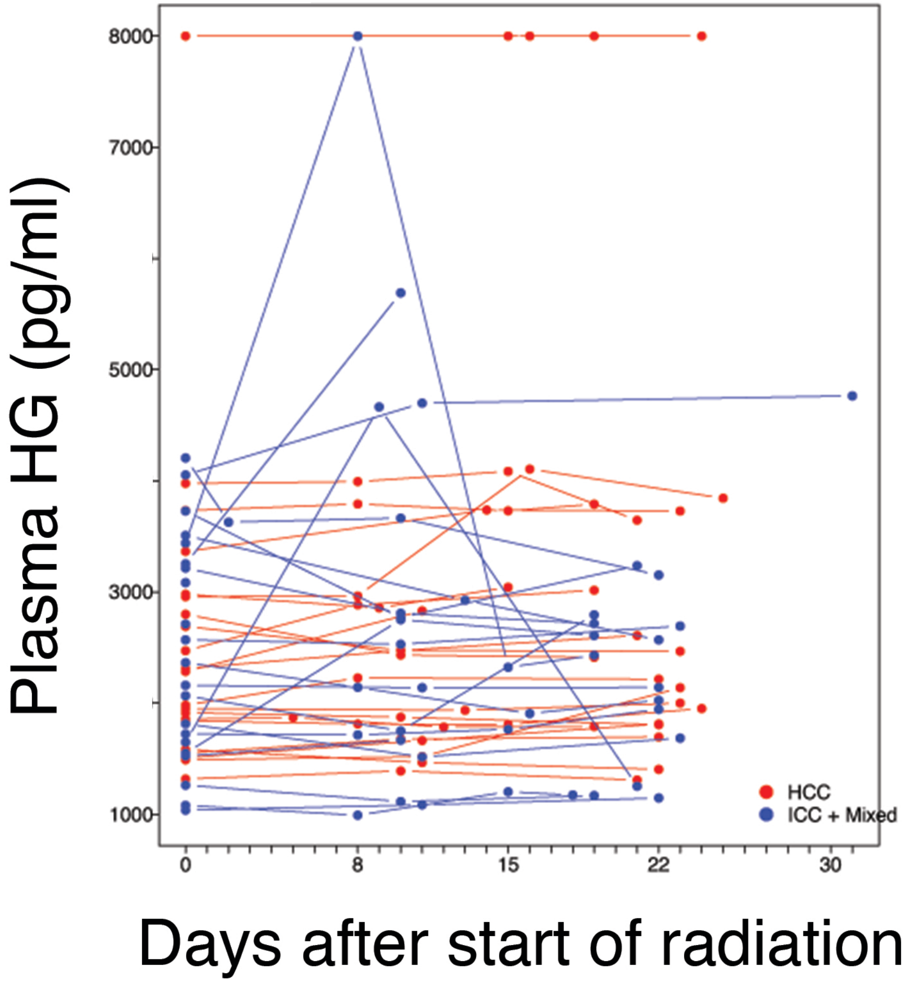

Supplemental Figure S1: Changes in plasma HGF during radiation therapy. No siggificant chages after proton therapy between pretreatment and day 22 (end of treatment). Data are shown individually for HCC and ICC patients.

Table S1 – Patient and Disease Characteristics				
		All Patients N=43	HCC N=22	ICC + Mixed N=21
Variable	Level	% (N) or Median (Range)	% (N) or Median (Range)	% (N) or Median (Range)
Demographics				
Age at Radiation Start Date		66 (36-88)	69 (54-88)	66 (36-82)
Sex	M	67.4% (29)	86.4% (19)	47.6% (10)
	F	32.6% (14)	13.6% (3)	52.4% (11)
Race	White	90.7% (39)	90.9% (20)	90.5% (19)
	Black	4.7% (2)	4.6% (1)	4.8% (1)
	Asian	2.3% (1)	0.0% (0)	4.8% (1)
	Unknown	2.3% (1)	4.6% (1)	0.0% (0)
Disease Characteristics				
Underlying Liver Disease	HCV (+/- EtOH)	39.5% (17)	59.1% (13)	19.1% (4)
	HBV	4.7% (2)	9.1% (2)	0.0% (0)
	EtOH	7.0% (3)	4.6% (1)	9.5% (2)
	NASH	4.7% (2)	4.6% (1)	4.8% (1)
	Other	4.7% (2)	0.0% (0)	9.5% (2)
	None	39.5% (17)	22.7% (5)	57.1% (12)
ECOG Performance Status	0	37.2% (16)	40.9% (9)	33.3% (7)
	1	60.5% (26)	54.6% (12)	66.7% (14)
	2	2.3% (1)	4.6% (1)	0.0% (0)
CTP	A or no Cirrhosis	86.0% (37)	86.4% (19)	85.7% (18)
	B	14.0% (6)	13.6% (3)	14.3% (3)
BCLC Stage	A-B	N/A	40.9% (9)	N/A
	C	N/A	59.1% (13)	N/A
CLIP Score	0-1	81.4% (35)	72.7% (16)	90.5% (19)
	2-3	18.6% (8)	27.3% (6)	9.5% (2)
Tumor Vascular Thrombosis	Yes	41.9% (18)	40.9% (9)	42.9% (9)
	No	58.1% (25)	59.1% (13)	57.1% (12)
Disease Status	Newly Diagnosed	97.7% (42)	95.4% (21)	100.0% (21)
	Locally Recurrent	2.3% (1)	4.6% (1)	0.0% (0)
Number of Nodular Tumors	1	79.1% (34)	72.7% (16)	85.7% (18)
	2	18.6% (8)	22.7% (5)	14.3% (3)
	3	2.3% (1)	4.6% (1)	0.0% (0)
Longest Tumor Dimension, cm		5.7 (2.2 – 12.0)	6.0 (2.6 – 12.0)	5.4 (2.2 – 10.9)
Sum of Longest Tumor Diameters, cm		6.5 (2.6 – 12.0)	7.1 (2.6 – 12.0)	5.4 (3.0 – 10.9)

Table S1 – Patient and Disease Characteristics				
		All Patients<br>N=43	HCC<br>N=22	ICC + Mixed<br>N=21
Variable	Level	% (N) or<br>Median (Range)	% (N) or<br>Median (Range)	% (N) or<br>Median (Range)
Biochemical Analysis				
Total Bilirubin, mg/dL		0.7 (0.2 – 3.2)	0.7 (0.2 – 2.8)	0.8 (0.2 – 3.2)
Platelets, k/UL		158 (59 – 335)	149.5 (61 – 259)	183 (59 – 335)
AFP, ng/mL		7.8 (1.3 – 66081)	55.7 (1.3 – 66081)	4.6 (1.3 – 90.9)
CA-19.9 (u/mL)*		46 (0 – 10549)	31 (0 – 311)	76 (0 – 10549)
Previous Therapy				
Any Surgical Resection	Yes	2.3% (1)	4.6% (1)	0.0% (0)
Any Transarterial Chemoembolization	Yes	7.0% (3)	13.6% (3)	0.0% (0)
Any Radiofrequency Ablation	Yes	4.7% (2)	9.1% (2)	0.0% (0)
Any Chemotherapy	Yes	32.6% (14)	9.1% (2)	57.1% (12)
None	Yes	53.5% (23)	63.6% (14)	42.9% (9)
* CA-19.9: N=41				

Table S2 – Treatment Characteristics			
	All Patients<br>N=43	HCC<br>N=22	ICC + Mixed<br>N=21
Variable	% (N) or<br>Median (Range)	% (N) or<br>Median (Range)	% (N) or<br>Median (Range)
GTV Volume, cm3	86.5 (3.7 – 500.6)	116.6 (9.9 – 500.6)	63.9 (3.7 – 310.9)
Whole Liver Volume, cm3	1701.6 (612.9 – 2790.3)	1756.9 (1195.8 – 2790.3)	1637.8 (612.9 – 2522.4)
Mean Liver Dose, GyRBE	18.3 (3.2 – 24.6)	19.3 (6.2 – 24.3)	15.8 (3.2 – 24.6)
Dose Delivered, GyRBE	58.0 (15.1 – 67.5)	58.0 (45.0 – 67.5)	58.0 (15.1 – 67.5)
Dose Completed	93.0% (40)	95.5% (21)	90.5% (19)

Table S3 – Patient Characteristics by Pretreatment HGF				
		< median N=21	≥ median N=22	
Variable	Level	% (N) or Median (Range)	% (N) or Median (Range)	p-value
Age at Radiation Start Date		69 (36-88)	65 (53-87)	0.326
Sex	M	66.7% (14)	68.2% (15)	1.000
	F	33.3% (7)	31.8% (7)	
Underlying Liver Disease	Present	47.6% (10)	72.7% (16)	0.124
	None	52.4% (11)	27.3% (6)	
ECOG Performance Status	0	38.1% (8)	36.4% (8)	1.000
	1-2	60.5% (13)	63.6% (14)	
CTP	A or no Cirrhosis	100.0% (21)	72.7% (16)	0.021
	B	0.0% (0)	27.3% (6)	
CLIP Score	0-1	19.5% (19)	72.7% (16)	0.240
	2-3	9.5% (2)	27.3% (6)	
Tumor Vascular Thrombosis	Yes	33.3% (7)	50.0% (11)	0.358
	No	66.7% (14)	50.0% (11)	
Number of Nodular Tumors	1	85.7% (18)	72.7% (16)	0.457
	2-3	14.3% (3)	27.3% (6)	
Longest Tumor Dimension, cm		4.7 (2.2 – 9.6)	6.8 (3.0 – 12.0)	0.088
Sum of Longest Tumor Diameters, cm		4.7 (2.6 – 10.4)	7.1 (3.0 – 12.0)	0.050
Total Bilirubin, mg/dL		0.6 (0.2 – 0.9)	1.0 (0.3 – 3.2)	0.005
Platelets, k/UL		191 (67 – 284)	125.5 (59 – 335)	0.066
AFP, ng/mL		6.0 (1.3 – 66081)	13.3 (1.3 – 41572)	0.216
CA-19.9 (u/mL)		25 (0 – 10549)	65 (0 – 2438)	0.080
Previous therapy	Any	47.6% (10)	45.4% (10)	1.000
	None	52.4% (11)	54.6% (12)	
Disease type	HCC	52.4% (11)	50.0% (11)	1.000
	ICC + Mixed	47.6% (10)	50.0% (11)	

Table S4 – Patient and Treatment Characteristics (n=101)			
Variable	Level	% (N) or Median (Range)	
Age		60 (54-64)	
Underlying Liver Disease	HBV	56.6% (56/99)	
	HCV	32.3% (32/99)	
	HDV	28.3% (28/99)	
Surgical Treatment	Liver Resection	43.6% (44/101)	
	Liver Transplantation	55.4% (56/101)	
	Synchronous	1.0% (1/101)	
Number of Nodular Tumors	1	67.0% (65/97)	
	2	19.6% (19/97)	
	3	9.3% (9/97)	
	4	3.1% (3/97)	
	7	1.0% (1/97)	
Longest Tumor Dimension, cm		4.7 ± 2.4 (91)	
Pre-operative Cirrhosis	Present	86.9% (86/99)	
	Absent	13.1% (13/99)	
Plasma HGF (pg/ml)	Median and interquartile range	No liver cirrhosis	Liver cirrhosis
		1,827 [1,386, 2,671]	4,612 [2,976, 6,656]

Supplemental Methods

Patients. We evaluated the impact of plasma HGF levels on overall survival (OS) and hepatic function after hypofractionated radiotherapy with protons for primary liver cancers in all 43—22 hepatocellular carcinoma (HCC), 20 intrahepatic cholangiocarcinoma (ICC) and 1 mixed HCC/ICC—patients enrolled at Massachusetts General Hospital (MGH), Boston, USA, in a prospective multi-site phase II clinical trial (NCT00976898) (**Tables S1-S3**).

Eligibility criteria in radiation therapy trial (reproduced from Hong et al., *J Clin Oncol* 2016) (3). Patients were enrolled in a prospective clinical trial (NCT00976898) approved by the institutional review boards of each of the participating institutions. Adult patients age 18 years or older were required to have biopsy-proven unresectable or locally recurrent HCC or ICC. Single or multinodular tumors (up to three) were permitted. Maximum tumor diameter permitted was 12 cm for solitary tumors, 10 cm if two tumors, and 6 cm if three tumors. Patients were required to have no evidence of extrahepatic tumor by computed tomography (CT) scan and an Eastern Cooperative Oncology Group (ECOG) performance status of 0 to 2. In patients with underlying cirrhosis, only a Child-Turcotte-Pugh (CTP) score of A or B was permitted. Adequate organ and marrow function was required and defined as absolute neutrophil count ≥750/mL; platelets ≥25,000/mL; total bilirubin ≤4 x 3 institutional upper limit of normal; transaminases ≤6 x 3 institutional upper limit of normal; and creatinine ≤2 mg/dL. No prior liver radiation, including radioembolization, was permitted. Patients were identified as having unresectable tumors after review with transplant surgery and surgical oncology physicians at the institutional multidisciplinary liver conference. The methods were performed in accordance with relevant guidelines and regulations and approved by the Institutional Review Boards of Dana Farber/Harvard Cancer Center, Boston, USA and Fundeni Clinical Institute, Bucharest, Romania. Written informed consent was obtained from all protocol patients before initiation of any study procedures.

Biomarker analysis. Worsening hepatic function was defined as an increase in CTP score by at least 1 point within 3 months. To delineate the impact of the therapeutic intervention on biomarker association, we also measured pretreatment plasma HGF levels in 101 patients with HCC who underwent surgical treatments, 86 of whom presented with underlying cirrhosis, at Fundeni Clinical Institute, Romania (**Table S4**). Plasma analysis of HGF concentration was carried out in duplicate in the CLIA-certified facility of the Steele Laboratories at MGH, using ELISA kits (R&D Systems).

In the radiotherapy study, progression-free survival (PFS) and overall survival (OS) were calculated from the first day of treatment. OS time was censored at the date of last follow-up for of patients still alive. PFS was measured until the earliest of any documented recurrence or death, and otherwise censored at the date of last follow-up. In the surgical study, we defined disease-free survival (DFS) as the time from surgery to any local tumor recurrence or distant-organ metastasis, and OS as the period of time from surgery until death or last follow-up.

Statistical analysis. Wilcoxon rank-sum test was used to compare the distribution of pretreatment plasma HGF levels between patients with and without an increase in CTP. Wilcoxon signed-rank test was used to assess the change in plasma HGF levels between baseline and day 8 or day 15. The Kaplan-Meier method was used to estimate 2-year OS, and the logrank test was used to compare OS and PFS between patients with high and low pretreatment plasma HGF. Data analysis was performed using SAS 9.4 (SAS Inst Inc, Cary, NC) and R 3.3.1 (R Foundation), with p-values based on a two-sided hypothesis. Circulating plasma HGF correlation with DFS or OS in patients undergoing surgical treatment was tested using Wald test in univariate Cox regression analysis, using log-transformed covariates. Comparison of biomarker levels after stratifying patients based on presence of cirrhosis was performed using exact Wilcoxon test, and correlation with Model for End-stage Liver Disease (MELD) score was evaluated using Spearman rank correlation test.
